# Multipole Modes Excitation of uncoupled dark Plasmons Resonators based on Frequency Selective Surface at X-band Frequency Regime

**DOI:** 10.1038/s41598-017-09845-3

**Published:** 2017-08-25

**Authors:** Yu Lan, Yuehang Xu, Yonghao Jia, Tengda Mei, Shiwei Qu, Bo Yan, Deqiang Yang, Bo Chen, Ruimin Xu, Yanrong Li

**Affiliations:** 1 School of electronic engineering, The University of Electronic Science and Technology of China, 611731 Chengdu, P.R. China; 2 State Key Laboratory of Electronic Thin Films and Integrated Devices, University of Electronic Science and Technology of China, 610054 Chengdu, P.R. China

## Abstract

In this report, we theoretically and experimentally demonstrates that multipole modes could be excited effectively in dark plasmonic resonators without introducing any other bright resonators at microwave range based on a two-dimensional frequency selective surface (FSS) structure. These excited multipole resonances are closely related to the coupling strength between adjacent S-LSPs resonators (the periodicity of the FSS). The modes splitting effects and resonance frequencies of the excited multipole modes are regulated by changing the numbers of grooves and inner disk radius, both of which play significant roles in the excitation of the dark S-LSPs disk resonator at normal incidence. Moreover, the multipole resonances characteristics of dark S-LSPs resonators in the case of oblique incidence are also presented. Observation of such multipole resonances in dark S-LSPs without introducing extra bright resonance at normal/oblique incidence would find more potential applications in microwave and terahertz based sensors, plasmonic resonance devices and metamaterial devices.

## Introduction

In recent years, the spoof local surface plasmons (S-LSPs) resonators based on planar corrugated metal disk have been proved their potentials to support electromagnetic field in planar microwave applications, resulting in burgeoning explorations of new microwave circuit structure and formations with exciting functionalities and properties. Surface plasmons (SPs) are highly localized surface electron density fluctuation located at the interface between two materials with opposite permittivities (e.g., metal and dielectric) which the field perpendicular to the surface decays exponentially with the distance from the surface, with the feature of strong field enhancement and confinement^1, 2^. In optical frequency range (visible and near-infrard regions) whose frequency is much higher than the collision frequency of metals (the order is 1014 GHz), the metal particle itself behaves as plasma with negative permittivity, leading to strong interaction coupling between electromagnetic wave and free charges in the metals. Typically, in optical frequency range, SPs are identified as two existent forms: either in the form of surface plasmon polaritons (SPPs) which are traveling along the metal-dielectric interface as propagating modes^3–5^ or localized surface plasmons (LSPs) as resonance mode^5–9^. However, at the microwave and terahertz regions whose frequencies are much lower than the collision frequency of metals, metal behaviors more akin to perfect electric conductor (PEC) instead of plasmas, resulting in a weak interaction force between electromagnetic waves and electron plasmas^10–12^ in metal particles. Instead of metal particle, corrugated metallic strip with subwavelength width^13^ and a spoof local surface plasmons (S-LSPs) resonators (periodic textured metallic disk structure)^14^ are theoretically presented to excite and support spoof SPPs and LSPs in microwave and terahertz frequency range, respectively. This planar periodic corrugated structure have the merit of compatibility and integration with the conventional microwave components and circuits, meaning that the microwave surface plasmons (SPs) circuits can be manufactured using the planar printed circuit board (PCB) technology. Since then, many new fundamental function microwave circuits based on SPs are exploited and demonstrated for SPPs^15–23^ and LSPs^24–28^.

The uncoupled S-LSPs resonator is also called as a dark multipole plasmonic resonator^29^, because that a uncouple S-LSPs resonator could only support dipole mode due to the perfect symmetry of the structure, instead of multipole modes. The existing excitation methods for dark S-LSPs resonators can be classified as two types: grazing incidence and normal incidence. Normal incidence is always used as the excitation source^29, 30^ in terahertz and optical frequency regime, but grazing incidence source is conventionally used in microwave frequency range^24–28^ which cannot be extend into the terahertz or optical range due to physical dimension limit. In the microwave frequency range, the reported researches of excitation methods for dark S-LSPs resonators are primarily concentrated on grazing incidence, which extra introduces another bright resonance (monopole antenna^25^ and Vivaldi antenna^31^) to induce multipole resonance modes in dark S-LSPs resonators. Actually, this combination excitation can be seen as an extension to the coupling and excitation mechanism of electromagnetically induced transparency (EIT) systems^32–34^ from the optical to microwave frequency regime. The monopole antennas in microwave function as the bright resonators which cut wire^35^ or C shape arm^29^ is typically used in optical and terahertz frequency of EIT systems.

Until now, there is no reports on multiple modes excitation in microwave dark S-LSPs resonators at normal/oblique incidence, which definitely would offer design advantages and flexibility in future microwave S-LSPs applications by taking advantage of the multipolar resonance property of S-LSPs resonator. In this report, the multipole modes excitation in dark S-LSPs resonator at X-band is studied in the case of grazing, normal and oblique incidence. A monopole antenna is introduced to induce multipole modes in S-LSPs resonator at grazing incidence and a microwave frequency selective surface consisting of dark S-LSPs resonators is theoretically and experimentally proposed at normal/oblique incidence. In previous work^29^, only the dipole mode, instead of multipole modes, was observed in terahertz dark S-LSPs resonators at normal incidence. Here we extend the dark S-LSPs resonance from terahertz to X-band (8G Hz–12 GHz) and construct a frequency selective surface consisting of tightly coupled S-LSPs resonators (the coupling length D = 0.03). Via the strong coupling between the adjacent S-LSPs resonators in the frequency selective surface, we firstly observe multipole modes excitation phenomenon in dark S-LSPs resonator without introducing extra bright resonators and obtain evident transmission dips corresponding to even resonance modes (dipole, hexapole mode and decapole mode) in X-band, which means that even multipole modes could be directly excited in this symmetric dark S-LSPs resonators at normal incidence. Then we investigate the unique features of the excited multipole modes in X-band by varying three parameters of the frequency selective surface: the periodicity (P) of FSS units, the grooves’ numbers (N) and the radius (r) of inner metallic rod. Finally, we reveal that the odd modes (quadrupooe and octupole modes) would also be excited in dark S-LSPs resonators by changing the normal incidence as oblique incidence and modes splitting effects occurs. Moreover, the frequency selective surface can acts as a selective electromagnetic switch at some special frequency bands under normal and oblique incidence condition. These findings of this report not only propose a new method to directly excite multipole modes in dark S-LSPs resonators at normal/oblique incidence in microwave, but also promise new supplementary applications in microwave and terahertz frequency regime based on S-LSPs resonators, such as point-to-point classified communication, switch, modulators and metamaterials devices.

## Results

### Grazing incidence in spoof local surface plasmons (S-LSPs) resonator

**Figure 1:**
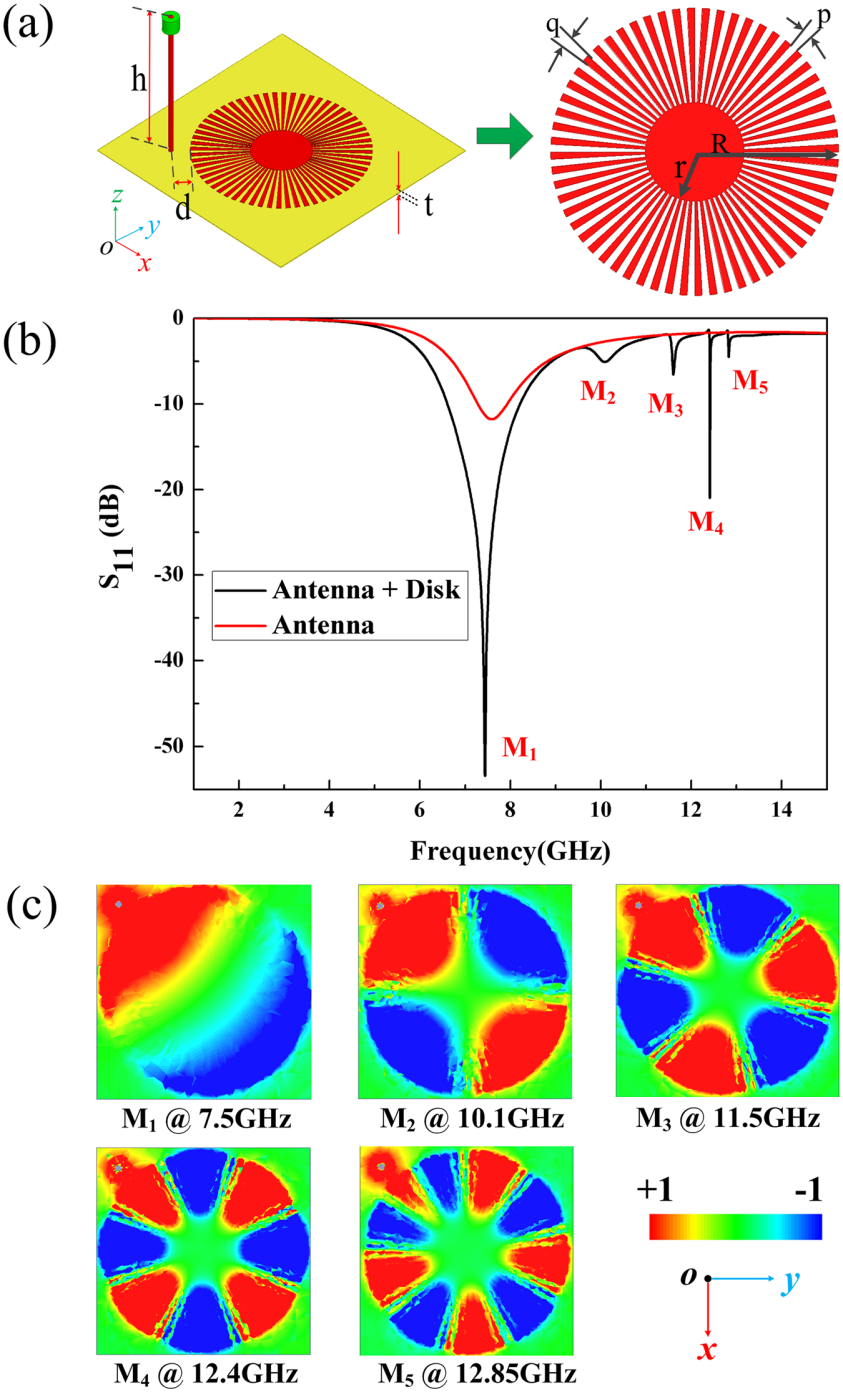
Multipole modes excitation in spoof local surface plasmons (S-LSPs) resonators excited by a monopoly antenna. (**a**) The schematic and geometry of S-LSPs printed on the Rogers ULTRLAM 3850 substrates excited by a monopoly antenna. The monopoly antenna with the height of h = 10.6 mm is placed d = 2 mm away from the left-side of the S-LSPs. (**b**) Simulated S11 of the single monopole antenna and the single S-LSPs resonator excited by the monopole antenna. (**c**) The simulated near field modes patterns of vertical electric field (Ez) on the plane 0.5 mm above the S-LSPs for the first five resonance modes: (M_1_) the dipole mode (even mode) at 7.5 GHz, (M_2_) the quadrupole mode (odd mode) at 10.1 GHz, (M_3_) the hexapole mode (even mode) at 11.5 GHz, (M_4_) the octopole mode (odd mode) at 12.4 GHz and (M_5_) the decapole mode (even mode) at 12.85 GHz.

Firstly, to determine the resonance frequency, we investigated the resonance characteristic under in-plane excitation (grazing incident) condition in a single S-LSPs resonator excited by a monopole antenna at X-band frequency range. In this report, a planar metallic spoof local surface plasmons (S-LSPs) resonator consists of an ultrathin corrugated metallic disk (shown in Fig. 1) is used, which is easy to integrate with the conventional planar circuits and systems. The spoof-plasmonic resonators are printed on a liquid crystal polymer (LCP) dielectric substrate (ULTRLAM 3850^36^) offered as a double copper clad laminate with constant relative permittivity of 2.9, substrate thickness of 50 µm and copper laminate thickness of 0.018 mm, using conventional standard printed circuit board (PCB) technology. The ultrathin corrugated metallic disk is made up by an inner metallic rod with radius of r = 2.4 mm and a set of outer grooves of radius of R = 7.0 mm. The numbers, width and gap of the grooves are N = 60, p = q = 3°, respectively. Here, we directly evaluate the resonances frequencies of different resonance modes (dipole, quadrupole, hexapole, octopole, and decapole mode) in the S-LSPs resonators using the commercial software High Frequency Structure Simulator with version of 2015 (ANSYS HFSS V.15). To get the grazing incidence wave, a monopoly antenna with the center frequency of 7.6 GHz (height h = 10.6 mm) is introduced to excite the dark S-LSPs resonator and placed at 2 mm away from the edge of the corrugated metallic disk resonator, as shown in the inset of Fig. 1. From the simulated reflection coefficient (S_11_) in Fig. 1(b), we can see that five resonance points (M_1_, M_2_, M_3_, M_4_ and M_5_) occur in the S-LSPs resonator. Figure 1(c) provides the visual modes mapping of the multipolar resonance modes in S-LSPs resonators with the near vertical electric fields (Ez) on an observation plane which is 0.5 mm above the textured disk: the dipole mode (M_1_) located at 7.5 GHz, the quadrupole mode (M_2_) located at 10.1 GHz, the hexapole mode (M_3_) located at 11.5 GHz, the octopole mode (M_4_) located at 12.4 GHz and the decapole mode (M_5_) located at 12. 85 GHz. Although the modes mappings are somehow arbitrary for different modes, they have a significant symmetry that if we invert the modes mapping about its center: either the mapping shapes of vertical electric field (Ez) are invariant (E(r) = E(−r)) which are called odd modes (quadrupole mode and octopole mode); or the mapping shapes turn into their own opposite (E(r) = −E(−r)) which are called even modes (dipole mode, hexapole mode and decapole mode). As a result, by introducing a monopole antenna, the grazing incidence wave can couple effectively to the S-LSPs resonator and excite different high order resonator modes in this dark symmetric S-LSPs resonator with the increasing of the operation frequency.

### Normal incidence in spoof local surface plasmons (S-LSPs) resonator

**Figure 2:**
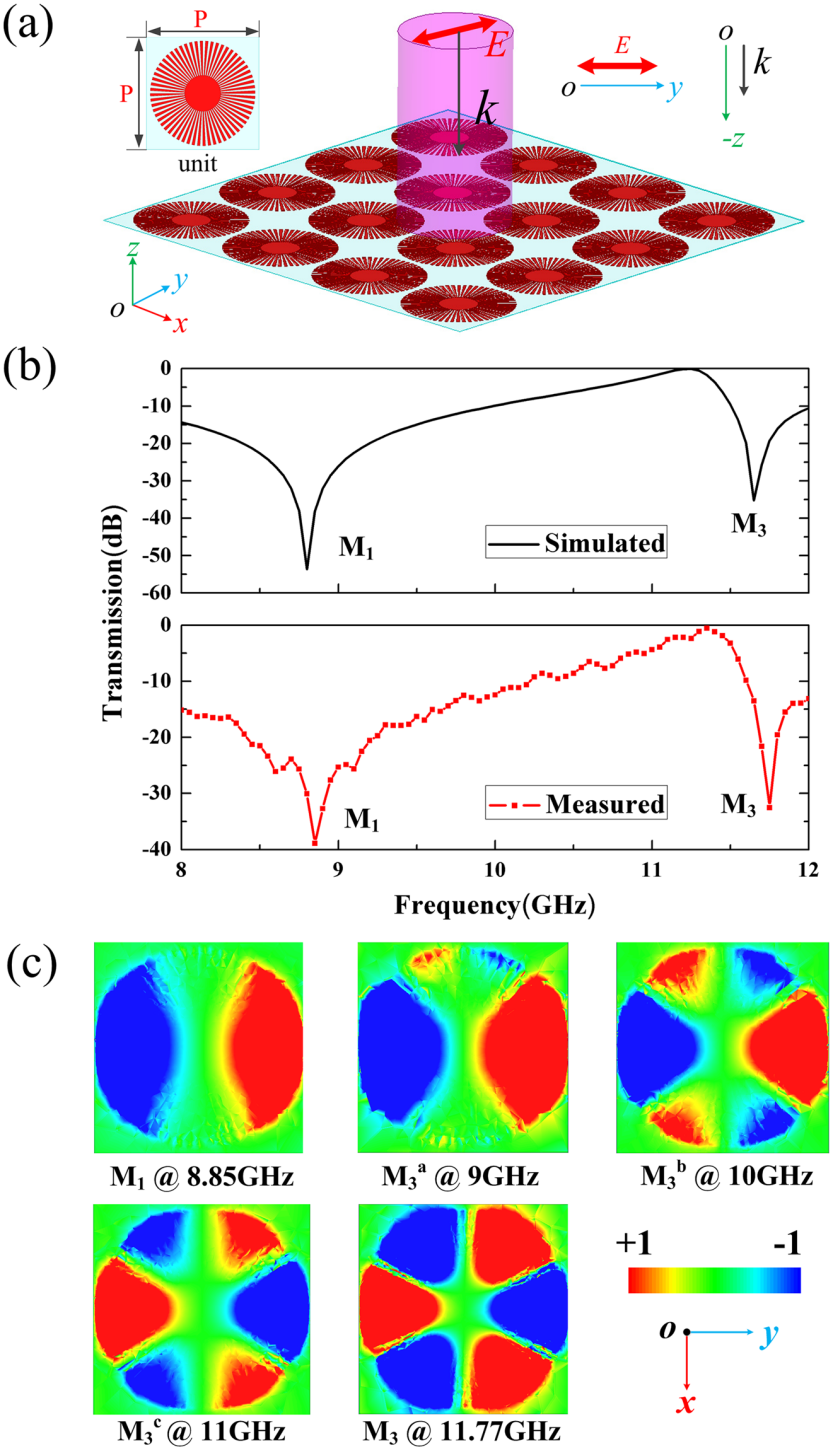
Multipole modes excitation in spoof local surface plasmons (S-LSPs) resonators excited by a normal incidence plane wave. (**a**) The schematics of the X-band frequency selective surface consist of spoof local surface plasmons (S-LSPs) resonators with the periodicity of P = 15 mm at normal incidence. The incidence wave vector lies along the z direction, with the electric field along the y direction. (**b**) Simulated and measured transmission spectra of the X-band frequency selective surface. (The simulated structure is infinite in x and y direction; the measured structure is a 225 units array with 15 rows and 15 lines in x and y direction). (**c**) The simulated near field modes patterns of vertical electric field (Ez) on the plane 0.5 mm above the S-LSPs: (M_1_) dipole mode at 8.85 GHz, (${{\rm{M}}}_{3}^{{\rm{a}}}$, ${{\rm{M}}}_{3}^{{\rm{b}}}$ and ${{\rm{M}}}_{3}^{{\rm{c}}}$) the transforming hexapole modes at 9 GHz, 10 GHz and 11 GHz, respectively, (M_3_) the hexapole mode at 11.77 GHz.

Fig. 1 indicates that the first three resonance modes (dipole (M_1_), quadrupole mode (M_2_) and hexapole mode (M_3_)) are located in X-band. Because that the dipole mode and quadrupole mode in S-LSPs resonators are the commonly modes which were used as the operation modes in microwave applications^24, 37^, so we emphatically study resonance characteristics of the first three modes in X-band frequency range at normal incidence. Fig. 2(a) shows the schematics of the proposed X-band frequency selective surface. The geometric parameters of units are chosen to be same as in Fig. 1(a), the periodicity is P = 15 mm, a plane wave act as incident excitation along the z direction with the E along the y direction. The master/slave boundaries and floquet ports which are always used in frequency selective surface simulation^38–41^ in HFSS are utilized to simulate the proposed frequency selective surface for extracting the transmission (S_21_) coefficient. The transmission measurement is obtained using an HP E8364C vector network analyzer connecting two X-band rectangle horn antennas (the measurement schematic and real test environment picture can be seen in Supplementary Fig. S1). Since the operating frequency of the horn antenna used in experiment is restricted at X-band, so the testing frequency range of all experiments is 8–12 GHz. The simulated structure has infinite elements in x and y direction; the measured structure is a prototype consisting of 225 units array with 15 rows and 15 lines in x and y direction which could offer enough accurate measurement results in most microwave frequency selective surface systems. The comparison of the simulated and measured results at normal is shown in Fig. 2(b), which shows that the experiment results are in good agreement with numerical predication. As shown in Fig. 2(b), the simulated (experiment) resonance frequency for dipole mode and hexapole mode are 8.8 GHz (8.85 GHz) and 11.65 GHz (11.75 GHz), respectively. To further reveal underlying physics of the multipolar resonances, the electric field *Ez* on the plane 0.5 mm above S-LSPs resonator corresponding to dips (M1 and M3) are presented in Fig. 2(c). From the Fig. 2(c), we can see that the dipole mode and hexapole mode correspond to the first two transmission dips shown in Fig. 2(b). But the dipole mode directly transfer to hexapole mode from the first transmission dip (dipole mode) to the second transmission dip (hexapole mode), which means that the quadrupole mode is suppressed in this dark S-LSPs resonator at normal incidence. As a result, though the quadrupole mode is suppressed, the frequency selective surface constructed by tightly coupled dark S-LSPs resonators can support multipole modes at normal incidence. Apparent multipole resonances (marked by M_1_, M_3_) could be found theoretically and observed experimentally as shown in Fig. 2(b).

**Figure 3:**
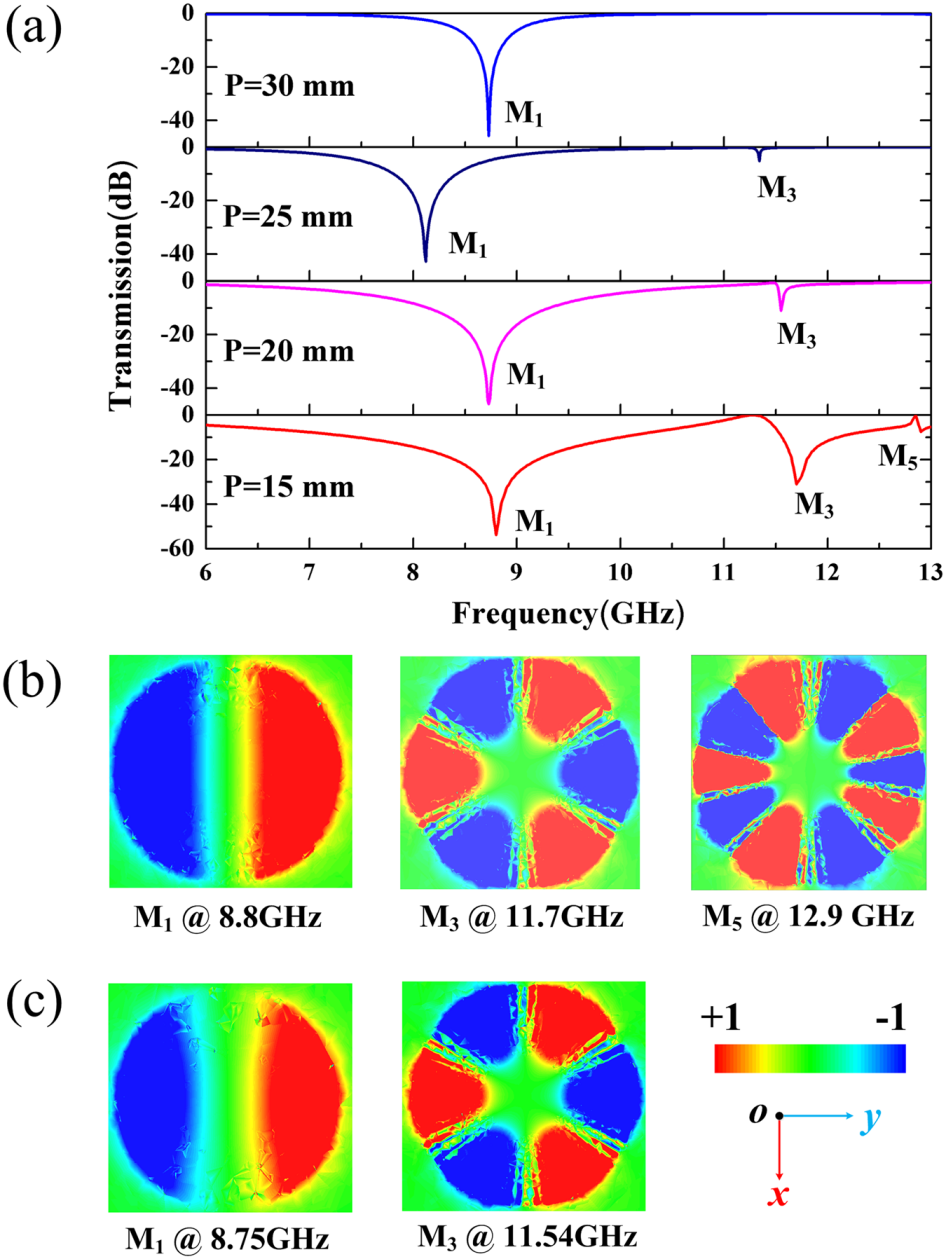
Multipole modes excitation in spoof local surface plasmons (S-LSPs) resonators excited by a normal incidence plane wave with respect to various periodicity (P = 30 mm, 25 mm, 20 mm and 15 mm). (**a**) Simulated transmission spectra for different periodicity. (**b**) The simulated near field modes patterns of vertical electric field (Ez) on the plane z = 0.5 mm above one unit S-LSPs resonators at the resonance points for P = 15 mm. (**c**) The simulated near field modes patterns of vertical electric field (Ez) for P = 20 mm.

To further investigate the excitation of multipole modes in dark S-LSPs resonance, the relationship between the first three modes (dipole, quadrupole and hexapole modes) and the coupling distance of the adjacent S-LSPs resonator are studied. We control the coupling distance by varying the periodicity (P) of the FSS. As shown in Fig. 3, when P = 40 mm, the coupling coefficient between two adjacent S-LSPs resonator is very small and it shows that the S-LSPs resonators only support a dipole resonator in the transmission spectrum. This results are accordance with the results in terahertz^29^. However, when P become smaller, the coupling coefficient is increased and the transmission dip effects of hexapole mode (M_3_) gradually become more obviously. Finally, when the P = 15 mm, the decapole mode (M_5_) appears. From the results in Fig. 3, we can see that the coupling distance dominates the multipole modes excitation in S-LSPs resonance. Moreover, at normal incidence, the excited modes are even modes (M_1_, M_3_ and M_5_) while the odd modes (M_2_ and M_4_) are suppressed by the perfect symmetric FSS structure, and the resonance frequency of the excited multipole modes (M_1_ and M_3_) have slight blue shift with increasing of the coupling coefficient(with the decrease of P).

**Figure 4:**
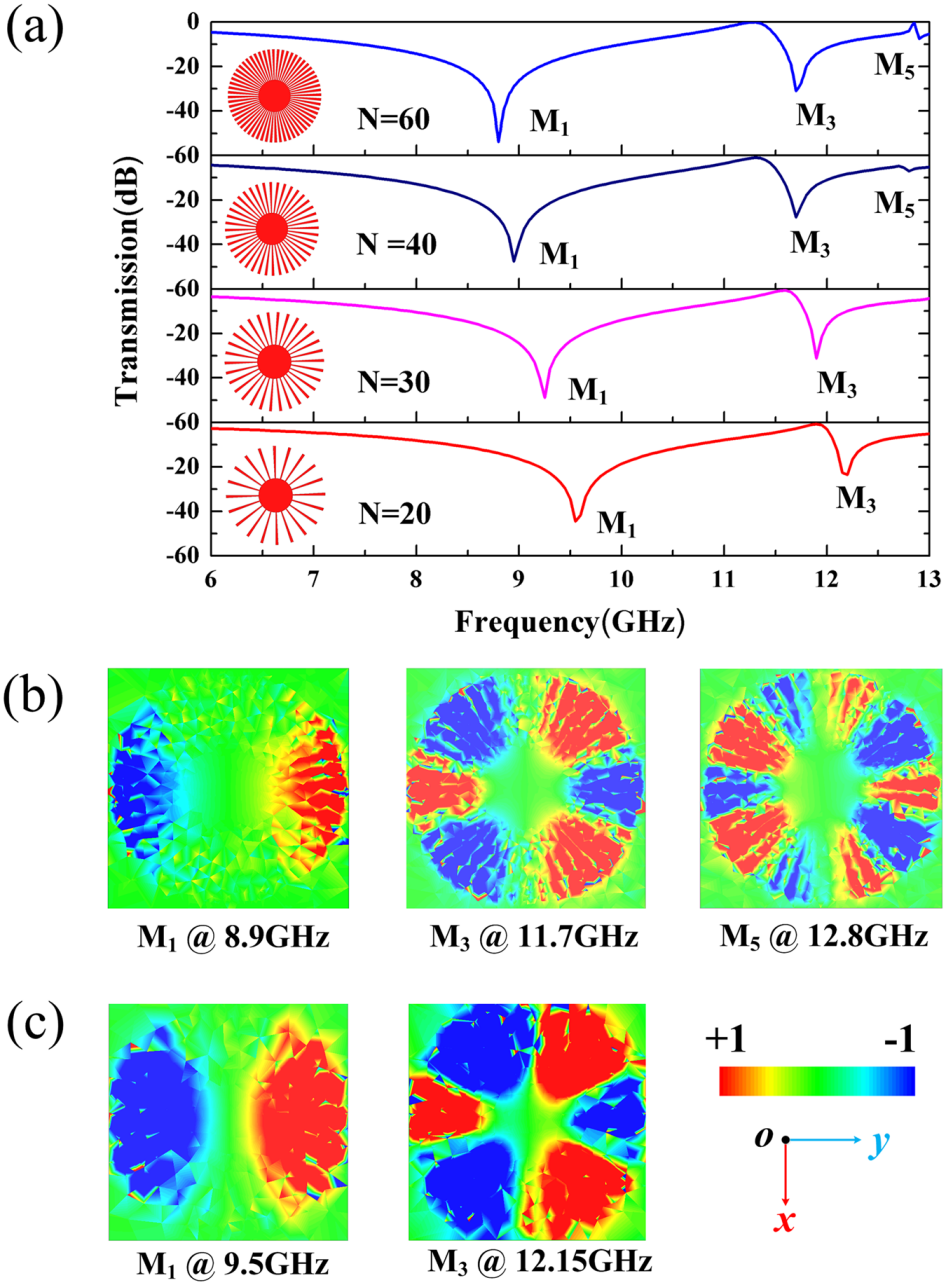
Multipole modes excitation in spoof local surface plasmons (S-LSPs) resonators excited by a normal incidence plane wave with respect to various grooves numbers (N = 60, 40, 30 and 20) with periodicity of P = 15 mm. (**a**) The simulated transmission spectra for various grooves numbers. (**b**) The simulated near field modes patterns of vertical electric field (Ez) on the plane z = 0.5 mm above one unit S-LSPs resonators at the resonance points for P = 15 mm and N = 40. (**c**) The simulated near field modes patterns of vertical electric field (Ez) at the resonance points for P = 15 mm and N = 20.

We can tune the resonance frequency of dark S-LSPs resonator at normal incidence by changing the grooves of S-LPSs disk, which can further affect the transmission dip depth and the positions of multipole modes in the transmission spectra. To illustrate this feature, the number of grooves of S-LSPs resonator is varied from 20 to 60 with a step of 10. The periodicity and the inner radius of the S-LSPs resonator is P = 15 mm and r = 2.4 mm, respectively. The transmission spectra of X-band FSS structure are plotted in Fig. 4(a) at different grooves numbers. When grooves number is decrease from N = 60 to N = 40, the transmission dips of resonance modes marked by M_1_ (dipole), M_3_ (hexapole) and M_5_ (decapole) are gradually decreased from −54 dB, −32 dB and −7 dB to −45 dB, −25 dB and −2 dB, respectively. With the decreasing of the grooves numbers from N = 60 to N = 20, the multipole resonance modes marked by M_1_ (dipole), and M_3_ (hexapole) will be shifted to higher frequency from 8.8 GHz and 11.7 GHz to 9.55 GHz and 12.2 GHz, respectively. As a result, with decreasing of the grooves numbers of S-LSPs resonator, the transmission dips would be more shallow and resonance frequency for multipole would have slight blue shift, as shown in Fig. 4(a).

**Figure 5:**
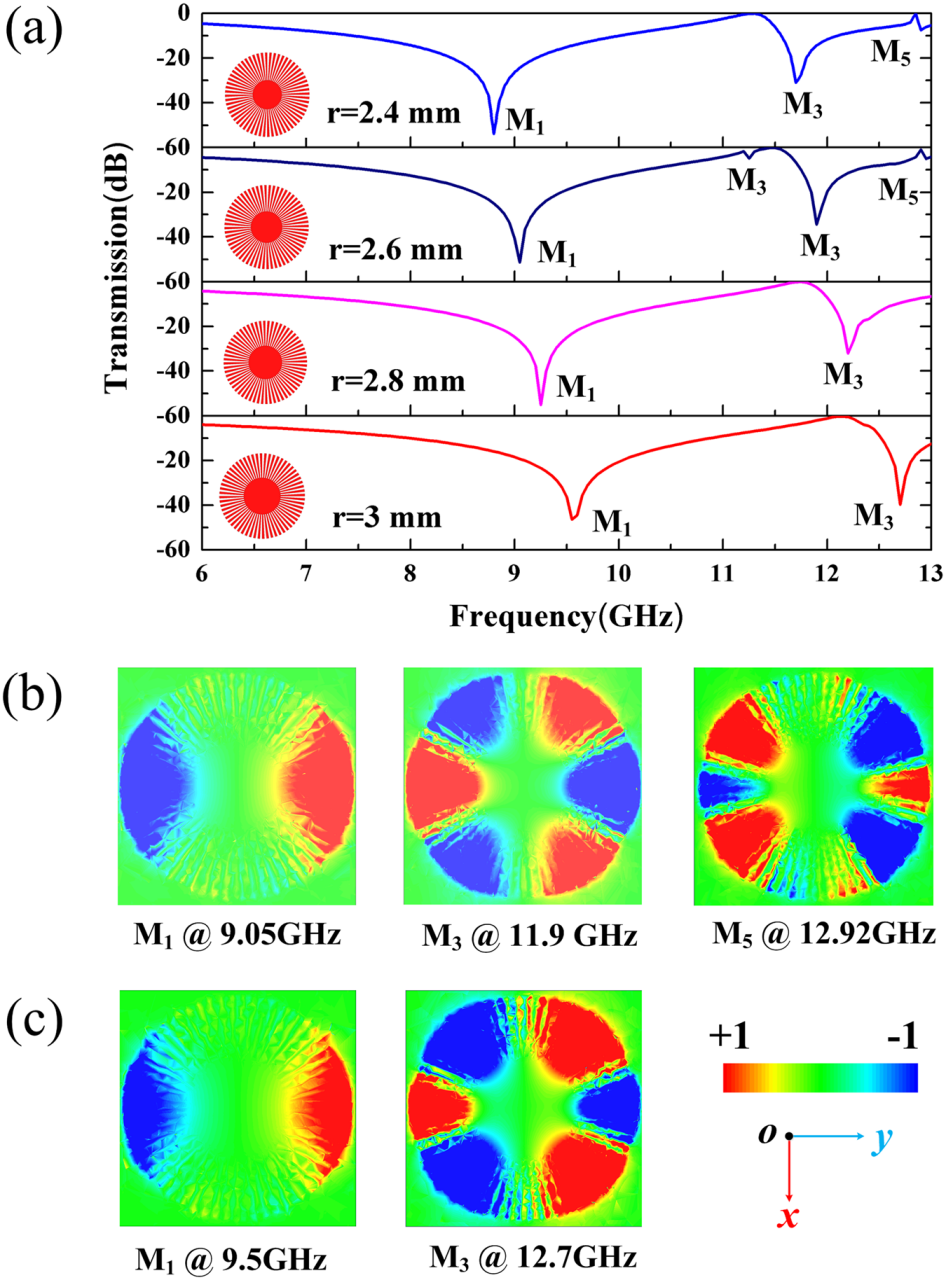
Multipole modes excitation in spoof local surface plasmons (S-LSPs) resonators excited by a normal incidence plane wave with respect to various inner radius (r = 2.4 mm, 2.6 mm, 2.8 mm and 3 mm) with periodicity of P = 15 mm. (**a**) Simulated Transmission spectra for various inner radius. (**b**) The near field modes patterns of vertical electric field (Ez) on the plane z = 0.5 mm above one unit S-LSPs resonators at the resonance points for P = 15 mm and r = 2.6 mm. (**c**) The near field modes patterns of vertical electric field (Ez) at the resonance points for P = 15 mm and r = 3 mm.

Next, the transmission spectra at different inner radii of S-LSPs resonators is studied. The simulated dispersion curves of the X-band FSS are shown in Fig. 5(a) with R = 7 mm, N = 60, p = q=3° and different radii (r=2.4 mm, 2.6 mm, 2.8 mm and 3 mm). In Fig. 5(a), with the increasing of inner radius, we can clearly obverse that blueshift phenomenon of the S-LSPs multipole modes frequency. When the inner radius is increased from 2.4 mm to 3 mm, the frequency of dipole (M1) mode and hexapole (M3) mode are increased from 8.8 GHz to 9.55 GHz, respectively. The vertical electric fields modes mapping of resonance frequencies for r = 2.6 mm (in Fig. 5(b)) show that hexapole (M_3_) mode exhibits mode splitting effect at 11.25 GHz and 11.9 GHz for r = 2.6 mm (the details about the difference of the vertical electric field hexapole mode (M3) mapping at 11.25 GHz and 11.9 GHz can be seen in Supplementary Fig. S2). As a consequence, the inner radius of S-LSPs resonators have significant influence on the excitation of multipole modes for plasmonic resonance, demonstrating the modes splitting effects for the multipole modes and blueshift for the resonance frequency.

### Oblique incidence in spoof local surface plasmons (S-LSPs) resonator

There is a fact that only the even multipole modes of dipole (M_1_), hexapole (M_3_) and decapole (M_5_) could be supported in the dark S-LSPs resonator at normal incidence, while the odd multipole modes of quadrupole mode (M_2_) and octopole mode (M_4_) are inhibited. Compared with the known electromagnetically induced transparency (EIT) systems in the optical^32–34^ and terahertz frequency^29^, their interaction mechanisms have some common points that two-dimensional periodic structure consisting of identical plasmonic resonance units and the normal incidence source are utilized to excite the experimental systems. However, there is an obvious different between previous EIT systems and our work in this report. In the previous plasmonics EIT systems^29, 32–34^, they all introduce an extra bright resonance to destroy the perfect symmetry of dark plasmonic resonators for multipole modes exciting, while the FSS structure we proposed is a perfect symmetric structure without introducing any bright resonance. Especially in ref. 29, the author firstly got all the multipole modes (even and odd modes) after introducing a C-shape bright resonance to the dark S-LSPs resonator which made the structure is asymmetrical; then they just got the even multipole modes after adding two identical C-shape resonance placed on the opposite of the dark S-LSPs resonator which recovered the symmetry of the structure. The results in ref. 29 are similar with the phenomenon we met in this report. So we assume that the asymmetry of the plasmonic resonance is the key point for the excitation of multipole modes in S-LSPs resonators.

**Figure 6:**
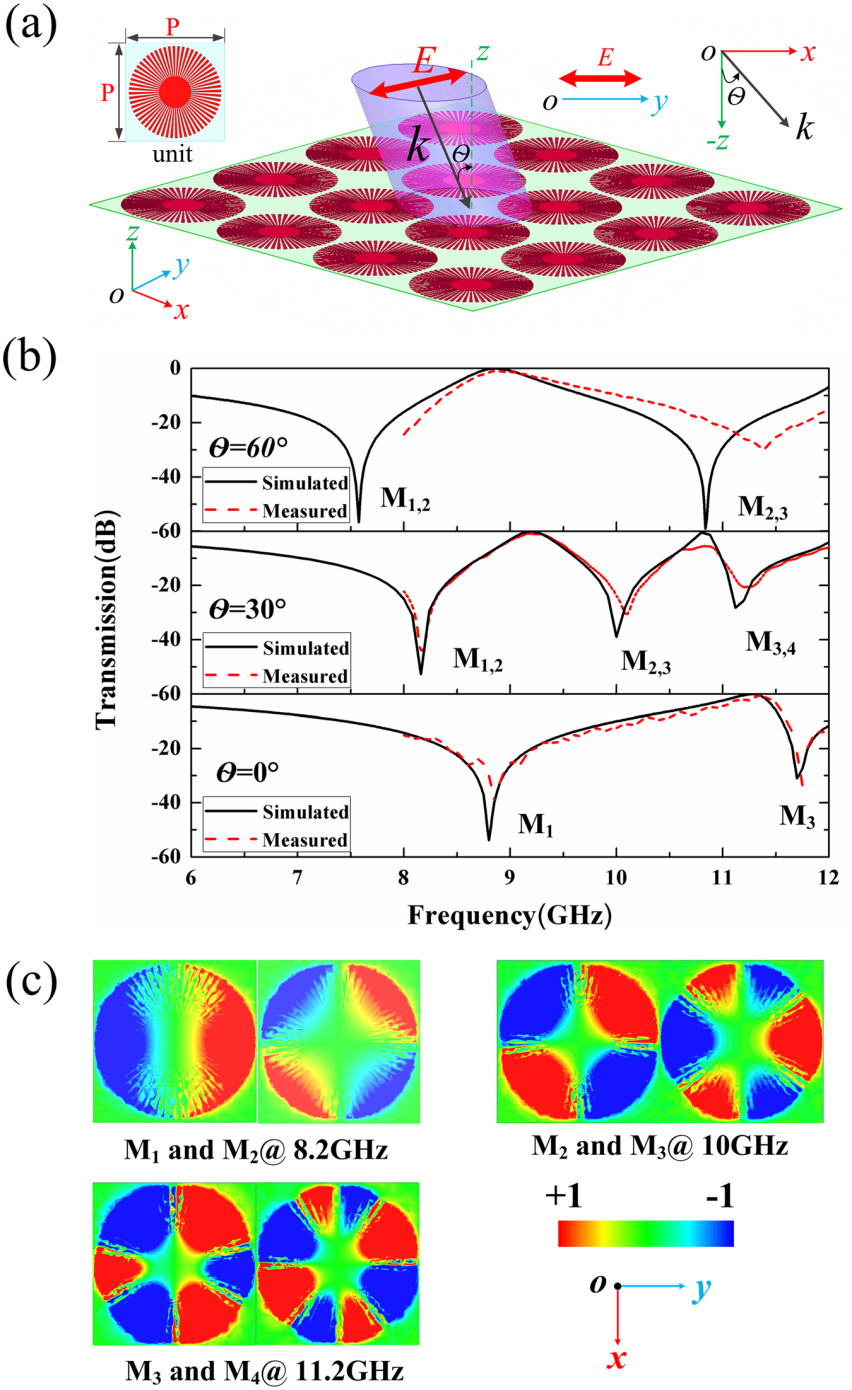
Multipole modes excitation in spoof local surface plasmons (S-LSPs) resonators excited by an oblique incidence plane wave with the periodicity of P=15 mm. (**a**) The schematics of the X-band frequency selective surface consist of S-LSPs resonators at oblique incidence. The incidence wave vector lie in the x-z plane and is titled angles of θ from the z direction, with the electric field along the y direction. (**b**) Simulated and measured transmission spectra of the X-band frequency selective surface with respect to various incidence angles (θ = 60°, θ = 30° and θ = 0°) with periodicity of P = 15 mm. (**c**) The near field modes patterns of vertical electric field (Ez) in the dark S-LSPs resonator at the transmission dips for P = 15 mm and θ = 30°: dipole (M_1_) and quadrupole (M_2_) modes at 8.2 GHz, quadrupole (M_2_) and hexapole (M_3_) mode at 10 GHz, and hexapole (M_3_) and octopole (M_4_) mode at 11.2 GHz.

To make the FSS structure sysytem asymmetry without introducing any other bright resonance, we change the normal incidence as oblique incidence. Figure 6(a) shows the schematics of FSS structure consisting of S-LSPs resonators at oblique incidence with periodicity of P = 15 mm. The incidence wave vector lies in the x-z plane and is titled angles of θ from the z direction, with the electric field along the y direction. Figure 6(b) shows the simulated and measured transmission spectra of FSS structure with respect of various incidence angles (θ = 60°, θ = 30°, and θ = 0°), where the incidence angle of θ = 0°is coincided with normal incidence. The experimental results fit well with the simulated results, which indicate that the simulated analysis would be reliable and rational for the future real applications. In Fig. 6(c), four multipole modes marked as dipole mode (M_1_), quadrupole mode (M_2_), hexapole mode (M_3_) and octopole mode (M_4_) could be excited at oblique incidence with incidence angle of θ = 30°. From the transmission spectra of Fig. 6(b), there are two obvious switchable frequency bands (located near at 8.8 GHz and 11.3 GHz) between the incidence angles of θ = 0°and θ = 60°. This unique feather is caused by the multipole modes excitation in S-LSPs resonance at oblique incidence, which would be used in switch and modulator devices based on S-LSPs resonator in the future. In addition, for θ = 30°and θ = 60°, some multipole modes have the modes overlapping effects which the dipole (M_1_) and quadrupole mode (M_2_), the quadrupole mode (M_2_) and hexapole mode (M_3_), hexapole mode (M_3_) and octopole mode (M_4_) would be excited simultaneously at the one frequency points hybridized in one cell; the mode splitting effect that one multiple mode would be excited at different frequency points at oblique incidence is also observed, which may be due to the tight coupling effect between S-LSPs resonators^24^. The resonance in transmission spectra at oblique incidence is much more complex that normal incidence. As a result, from the Fig. 6, we can see that the multipole modes (even and odd modes) could be both excited in the dark S-LSPs resonator at oblique incidence, and the multipole modes overlaping and splitting effects occur in S-LSPs resonators which could not be happened at normal incidence.

## Discussion

In summary, the multipole modes excitation of dark S-LSPs resonators in X-band is investigated at grazing incidence, normal incidence and oblique incidence conditions. At grazing incidence, a monopole antenna,which acts like a bright resonance in conventional EIT systems, was introduced to induce the multipole modes in S-LSPs, and both even and odd multipole modes were excited at S-LSPs resonators. Then, we proposed an X-band frequency selective surface (FSS) consisting of only dark S-LSPs resonators without introducing any other bright resonators to investigate the multipole modes excitation in dark S-LSPs resonators at normal and oblique incidence, which we also demonstrate the multipole modes excitation. For verifying the rationality of analysis, we experimentally obtain the transmission spectra at normal and oblique incidence, and the measured results fit well with the simulated result. The using of only dark S-LSPs resonators without introducing bright resonance made the whole structure is perfect symmetry, leading to polarization independence in such coupled structure which is another superiority compared with conventional EIT systems. Since the multipole modes excitation in dark S-LSPs resonator at normal and oblique incidence is much more flexible than grazing incidence case, our demonstrated structure may incubate more microwave and terahertz promising applications in multi-channel communication, sensors and metamaterial devices based on S-LSPs resonance.

## Methods

### Simulation

The numerical simulations are calculated by using the commercial electromagnetic simulator High Frequency Structure Simulator with version of 2015 (HFSS v.15). The simulation reflection coefficient (S_11_) of grazing incidence is based on driven mode, where only one monopole antenna and an S-LSPs resonator are used. The simulation transmission spectra of normal and oblique incidence are based on driven mode (network analysis), the master/slave boundary condition and the floquet ports are used. The electric distribution (Ez) mapping pattern are obtained on the plane 0.5 mm on the S-LSPs resonator.

### Sample fabrication and Experiment setup

All samples are fabricated on the 0.05 mm thick Rogers 3850 (Permittivity ε = 3.0; Loss tangent Δδ = 0.0025) printed on circuit boards with one side covered by 18 μm thick textured copper disks etched based on the designs, using traditional photolithography. The simulated Cu is default conductivity of 5.88 × 10^7^ S.m^−1^. Two X-band rectangle horn antennas working at 8–12 GHz are used to connect with two ports of an HP E8364C vector network analyzer to obtain the transmission spectra of normal and oblique incidence cases.

## Electronic supplementary material

Multipole Modes Excitation of uncoupled dark Plasmons Resonators based on Frequency Selective Surface at X-band Frequency Regime
