# Supplementary material for: Multipole Modes Excitation of uncoupled dark Plasmons Resonators based on Frequency Selective Surface at X-band Frequency Regime
Source: Sci Rep. 2017 Aug 25;7:9492. doi: 10.1038/s41598-017-09845-3 (PMC5573369; doi:10.1038/s41598-017-09845-3)
Supplement: Supplementary file 1 — Multipole Modes Excitation of uncoupled dark Plasmons Resonators based on Frequency Selective Surface at X-band Frequency Regime [file 41598_2017_9845_MOESM1_ESM.pdf]

Multipole Modes Excitation of uncoupled dark Plasmons Resonators based on Frequency Selective Surface at X-band Frequency Regime

Yu Lan, Yuehang Xu*, Yonghao Jia, Tengda Mei, Shiwei Qu, Bo Yan, Deqiang Yang, Bo Chen, Ruimin Xu, Yanrong Li

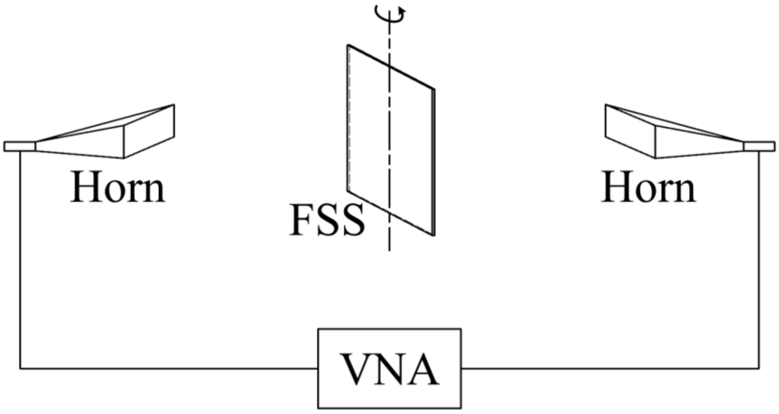

(a)

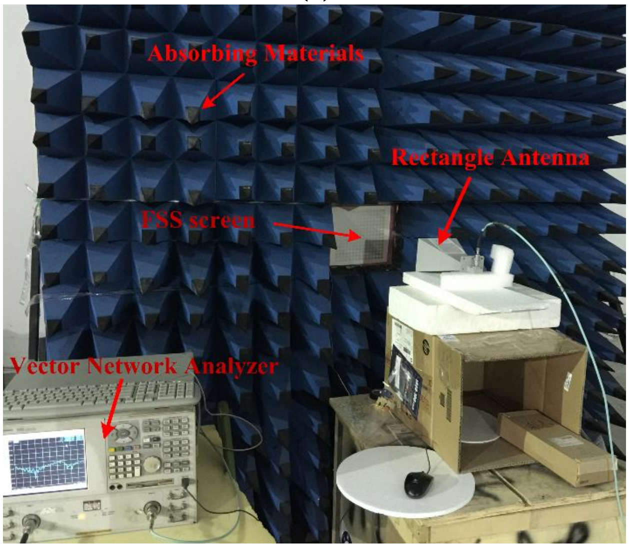

(b)

Fig.S1. (a) The measurement setup of the frequency-selective surface. (b)The real measurement environment.

The measurement results was obtained using an HP E8364C vector network analyzer connecting two rectangle horn antennas in a transmission measurement setup. Fig.S1 (a) shows the schematic of the transmission measurement setup. Fig.S1 (b) shows the real measurement environment. The reasons for

using the rectangle horn antenna is that the polarization of the radiation field at the far field could be accurately identified, because the excitation of the rectangle antenna is the TE_{10} mode for antenna feeding waveguide. Since the operating frequency range of rectangle horn antennas in experiment were restricted in X-band, so the testing was carried out in the frequency from 8GHz to 12GHz for getting more dependable measurement results.

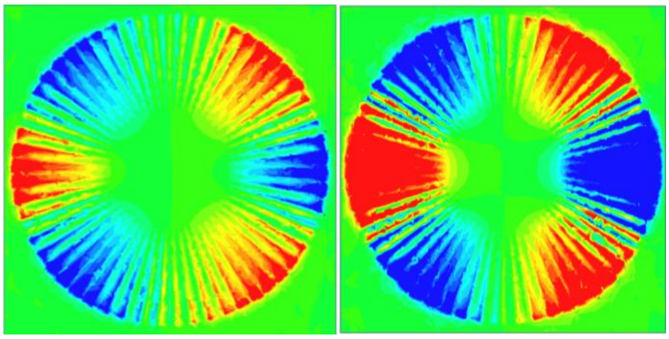

（a）M3@11.25GHz （b）M3@11.9GHz

Fig.S2. The near field modes patterns of vertical electric field (Ez) on the plane z=0.5 mm above one unit S-LSPs resonators at the resonance points for P=15 mm and r=2.6 mm. (a) hexapole mode (M3) mapping at 11.25GHz. (b) hexapole mode (M3) mapping at 11.9GHz.

Fig.S2 is the difference of the vertical electric field hexapole mode (M3) mapping at 11.25GHz and 11.9GHz. From the mapping of the hexapole mode (M3) at 11.25GHz and 11.9GHz, we can see that there is no sign of modes transfer. The mode splitting effects have been reported in the manuscript's reference [29] when changing the inner radius of the disk resonance in the case of normal incidence. But in [29], the splitting modes appeared at hexapole mode (M3) for r=75um and decapole mode (M5) for r=90um, respectively. These phenomenons indicate that the inner radius (r) plays significant roles in modes splitting effects in the spoof local surface plasmons (S-LSPs) resonators. The inner radius (r) not only impacts on the resonance frequency shift in the S-LSPs resonators, but also decides whether the modes splitting effects appear and which multipole modes appear.

[29]. Chen, L. et al. Excitation of dark multipolar plasmonic resonances at terahertz frequencies. Sci. Rep. 6, 22027; doi: 10.1038/srep22027 (2016).
